# Associations Between the Macular Microvasculatures and Subclinical Atherosclerosis in Patients With Type 2 Diabetes: An Optical Coherence Tomography Angiography Study

**DOI:** 10.3389/fmed.2022.843176

**Published:** 2022-03-04

**Authors:** Jooyoung Yoon, Hyo Joo Kang, Joo Yong Lee, June-Gone Kim, Young Hee Yoon, Chang Hee Jung, Yoon Jeon Kim

**Affiliations:** ^1^Department of Ophthalmology, Asan Medical Center, College of Medicine, University of Ulsan, Seoul, South Korea; ^2^Asan Diabetes Center, Asan Medical Center, Seoul, South Korea; ^3^Department of Internal Medicine, Asan Medical Center, College of Medicine, University of Ulsan, Seoul, South Korea

**Keywords:** carotid ultrasonography, optical coherence tomography angiography (OCTA), retinal microvasculatures, subclinical atherosclerosis, type 2 diabetes

## Abstract

**Objective:**

To investigate the associations between the macular microvasculature assessed by optical coherence tomography angiography (OCTA) and subclinical atherosclerosis in patients with type 2 diabetes.

**Methods:**

We included patients with type 2 diabetes who received comprehensive medical and ophthalmic evaluations, such as carotid ultrasonography and OCTA at a hospital-based diabetic clinic in a consecutive manner. Among them, 254 eyes with neither diabetic macular edema (DME) nor history of ophthalmic treatment from 254 patients were included. The presence of increased carotid intima-media thickness (IMT) (>1.0 mm) or carotid plaque was defined as subclinical atherosclerosis. OCTA characteristics focused on foveal avascular zone (FAZ) related parameters and parafoveal vessel density (VD) were compared in terms of subclinical atherosclerosis, and risk factors for subclinical atherosclerosis were identified using a multivariate logistic regression analysis.

**Results:**

Subclinical atherosclerosis was observed in 148 patients (58.3%). The subclinical atherosclerosis group were older (*p* < 0.001), had a greater portion of patients who were men (*p* = 0.001) and who had hypertension (*p* = 0.042), had longer diabetes duration (*p* = 0.014), and lower VD around FAZ (*p* = 0.010), and parafoveal VD (all *p* < 0.05). In the multivariate logistic regression analysis, older age (*p* ≤ 0.001), male sex (*p* ≤ 0.001), lower VD around FAZ (*p* = 0.043), lower parafoveal VD of both superficial capillary plexus (SCP) (*p* = 0.011), and deep capillary plexus (DCP) (*p* = 0.046) were significant factors for subclinical atherosclerosis.

**Conclusion:**

The decrease in VD around FAZ, and the VD loss in parafoveal area of both SCP and DCP were significantly associated with subclinical atherosclerosis in patients with type 2 diabetes, suggesting that common pathogenic mechanisms might predispose to diabetic micro- and macrovascular complications.

## Introduction

Carotid artery stenosis, an important, potentially life-threatening consequence of systemic atherosclerotic disease in the aging population, is responsible for 10–20% of the ischemic strokes, which are the second most common cause of death worldwide. Diabetes mellitus (DM), one of the major risk factors of carotid artery stenosis, results in systemic vascular complications: macro- and microvascular complications (1). Therefore, screening for the vascular abnormalities and prevention of irrecoverable damage in the high-risk patients are crucial to reduce the social and financial burden of DM (2). Traditionally, the macro- and microvascular complications of diabetes have been considered as the distinct and independent disorders. Recently, however, pathophysiological evidence and epidemiologic evidence suggest that these vascular complications may share common pathophysiological mechanisms (3).

Optical coherence tomography angiography (OCTA) is a new, non-invasive technology that enables the reproducible, quantitative assessment of the microcirculation of different retinal capillary layers (4–8). Unlike the fluorescein angiography, OCTA does not require intravenous dye to assess the retinal vasculature, and therefore causes less discomfort and pain, and is free from the potential systemic adverse effects (9). Characteristic retinal vascular alterations in OCTA have been well-described in patients with diabetic retinopathy (DR) from their early stage of diseases, and several reports showed that these changes were detectable even before the development of DR (10, 11). Recently, the clinical implications of the OCTA parameters for assessing associations with the carotid stenosis were investigated (12). However, not only carotid intima media thickness (IMT), but carotid plaque burden is also reported as a surrogate of atherosclerosis and predictor of future atherosclerotic cardiovascular diseases (13). Thus, we aimed to compare the retinal microvascular changes measured with OCTA in patients with type 2 diabetes in terms of the presence of carotid artery disease detected by carotid ultrasonography (US), the early indicator of systemic subclinical atherosclerosis (14). In addition, systemic and ophthalmologic factors related to subclinical atherosclerosis were evaluated.

## Methods

The research adhered to the tenets of Declaration of Helsinki. The study was approved by the international research board of Asan Medical Center (IRB No. 2020-0014). Informed consent was waived due to the retrospective nature of the study.

### Study Subjects

Patients with type 2 DM who received comprehensive medical and ophthalmic evaluations during the period from January 2017 to December 2019 at a hospital-based diabetic clinic (Asan Medical Center, Seoul, Korea) were selected by medical record review in a consecutive manner. Patients underwent vascular evaluation, such as carotid US, and ophthalmic evaluation, including OCTA at regular intervals based on their medical status, and those who had both carotid US and OCTA within 6 months interval were included in this retrospective observational study. We excluded patients if they had history of ophthalmic treatment, diabetic macular edema (DME), with concomitant ocular disease other than DR, or history of ocular trauma. For image qualities, those with poor OCTA quality, with a scan quality of 6 or less out of 10, were excluded. In addition, to minimize the possible errors in image analysis, proper segmentation without errors, and removal of projection artifact are carefully considered. When both eyes met the inclusion criteria, we included the right eye, and when only one eye of the two eyes satisfied the inclusion criteria, the corresponding eye was included in the study to include one eye for each patient.

At the initial visit of endocrinology, every patient underwent detailed medical and surgical history, such as medication information and duration for diabetes and hypertension, smoking habits, and alcoholic intake. In addition, at baseline and every visit, arterial blood pressure (BP), body weight, and height were measured, and body weight and height were used to calculate the body mass index (BMI), which was used for analysis. After overnight fasting, early morning blood samples were obtained and underwent a central, certified laboratory analysis. Measurements included were hemoglobin A1C (HbA1c), serum glucose level, several lipid parameters, and creatinine. HbA1c was measured using high-performance liquid chromatography (HPLC) of a Variant II Turbo (Bio-Rad Laboratories, Hercules, CA, USA). Fasting total cholesterol, high-density lipoprotein-cholesterol (HDL-C), low-density lipoprotein-cholesterol (LDL-C), and triglyceride (TG) were measured by using an enzymatic colorimetric method (Toshiba Medical Systems). Creatinine was measured by using the Jaffe method, and estimated glomerular filtration rate (eGFR) was calculated with the modified Modification of Diet in Renal Disease (MDRD) equation. In addition, urine tests were performed and urinary albumin-to-creatinine ratio (UACR) was calculated to determine the severity of albuminuria, using a photometric method of the Integra 800 system (Roche Diagnostics, Indianapolis, IN, USA) in a random spot urine collection.

At their initial visit and at each visit to a retina clinic, all patients underwent a comprehensive ophthalmologic examination that included a review of their ophthalmologic history, measurement of visual acuity, slit lamp biomicroscopy, and funduscopic examinations through dilated pupils by retinal specialists. The severity of DR was classified into 5 grades by the following criteria of the Early Treatment Diabetic Retinopathy Study (ETDRS): (1) no diabetic retinopathy—“no DR”; (2) mild non-proliferative diabetic retinopathy—“mild NPDR”; (3) moderate non-proliferative diabetic retinopathy—“moderate NPDR”; (4) severe non-proliferative diabetic retinopathy—“severe NPDR”; and (5) proliferative diabetic retinopathy—“PDR.”

#### Optical Coherence Tomography Angiography

The RTVue XR Avanti (Optovue, Fremont, CA, USA) spectral-domain OCT device with phase 7 AngioVue software was used for the OCT and OCT angiography examination. A 3 mm × 3-mm macular scans centered on the fovea were acquired. Each OCTA en face image contains 304 × 304 pixels created from the intersection of the 304 vertical and the 304 horizontal B-scans. AngioVue software automatically segments the B-scan images into four layers: superficial capillary plexus (SCP), deep capillary plexus (DCP), outer retina, and choriocapillaris layer. The SCP layer was segmented with an inner boundary set at 3 μm beneath the internal limiting membrane and an outer boundary at 15 μm beneath the inner plexiform layer. The DCP layer was segmented with an inner boundary set at 15 μm beneath the inner plexiform layer and an outer boundary at 70 μm beneath the inner plexiform layer. Using SCP and DCP images, following parameters were measured with the integrated automated software. For FAZ related parameters, area (mm^2^) and perimeter (mm) were measured and acircularity was calculated using those two parameters. In addition, vessel density (VD) around 300 μm boundary around FAZ and VD of each selected region (foveal and parafoveal area of four quadrants) were calculated as the percentage of area occupied by flowing blood vessels and was analyzed in both SCP and DCP, respectively.

#### Carotid Ultrasonography

Carotid artery examination was performed by a single specialized technician with patients in the supine position with the head elevated to 45 degrees and tilted to either side by 30 degrees and the operator seated at the head bed. High resolution ultrasound (HD 11 XE, Philips Healthcare, Andover MA) equipped with a high-frequency (5–12.5 MHz) linear transducer was used to acquire images of the left and right common carotid arteries. Carotid IMT scanning and reading was evaluated with the criteria of Mannheim Carotid Intima-Media Thickness Consensus (15). IMT was measured from the media-adventitia interface to the intima-lumen interface at the level of ~0.5 cm below the carotid-artery bulb, over a 1-cm segment of the artery, and the degree of stenosis was assessed. The value obtained through a QLAB IMT-quantification software measurement plug-in (Philips Healthcare) was used in analysis (16). The upper normal limit of IMT was 1.0 mm, and focal lesions with increased carotid IMT (>1.0 mm) or the presence of carotid plaque was defined as subclinical atherosclerosis (17).

#### Statistical Analysis

The following variables were analyzed in each patient: (i) demographic variables (i.e., age, sex, comorbidities with hypertension or hyperlipidemia, DM duration, and DM treatment), (ii) laboratory variables (i.e., carotid IMT, presence of carotid plaque, HbA1C, glucose, systolic BP (SBP) and diastolic BP (DBP), total cholesterol, TG, HDL and LDL-cholesterol, UACR, creatinine, and eGFR), (iii) ocular characteristics (i.e., BCVA and DR severity), and (iv) OCTA parameters (FAZ related parameters; area, perimeter, acircularity, and VD around FAZ, foveal and parafoveal VD in SCP and DCP).

Descriptive statistics were demonstrated in numbers and percentages for categorical variables and mean ± SD of continuous variables to present the baseline characteristics of study subjects. For comparison in terms of the presence of subclinical atherosclerosis (the subclinical atherosclerosis group and the non-subclinical atherosclerosis group), independent *t*-test or Mann–Whitney *U*-test was used depending on the normality of their distribution. Chi-squared test was used to compare the categorical data. To explore the factors significantly associated with subclinical atherosclerosis, logistic regression analyses were conducted. Univariate analyses were separately performed for each variable and those with *p* < 0.1 were included in the multivariate analysis with the forward elimination process. Odds ratios (*OR*s) with 95% *CI*s were calculated. All statistical analyses were performed using SPSS version 21.0 software (SPSS Inc., Chicago, IL, USA).

## Results

Of a total of 254 patients included in this analysis, 148 patients (58.3%) had subclinical atherosclerosis. Patients with subclinical atherosclerosis were older than those without (60.1 ± 9.1 vs. 54.1 ± 11.2 years, *p* < 0.001). Baseline characteristics in this study are summarized in Table 1. Patients with subclinical atherosclerosis had greater portion of male sex (72.3 vs. 52.9%, *p* = 0.001), hypertension (50.7 vs. 37.7%, *p* = 0.041), and longer duration of type 2 DM (19.4 ± 8.3 vs. 16.9 ± 7.6 years, *p* = 0.013). All the study participants were receiving either oral hypoglycemic agents or insulin injection or both, and the proportion of patients on insulin treatment and smoking status were not significantly different between the two groups. HbA1C, serum glucose, SBP, DBP, UACR, creatinine, and eGFR were not different between the two groups.

**Table 1:** Baseline demographics and clinical characteristics of patients in this study.

	**Total (*n* = 254)**	**Subclinical atherosclerosis (–) (*n* = 106)**	**Subclinical atherosclerosis (+) (*n* = 148)**	***P-*value**
Age (year)	57.6 ± 10.4	54.1 ± 11.2	60.1 ± 9.1	<0.001
Sex (male: female)	162: 92	55: 51	107: 41	0.001
Hypertension [*n* (%)]	115 (45.3)	40 (37.7)	75 (50.7)	0.041
DM duration (yr)	18.3 ± 8.1	16.8 ± 7.6	19.4 ± 8.3	0.013
DM treatment [*n* (%)]				0.630
OHA only	166 (65.4)	66 (62.3)	100 (67.6)	
Insulin	88 (34.5)	40 (37.7)	48 (32.4)	
Hyperlipidemia [*n* (%)]	141 (55.5)	52 (49.1)	89 (59.7)	0.256
Smoking status [*n* (%)]				0.325
Non-smoker	134 (52.8)	61 (57.5)	73 (49.3)	
Ex-smoker	69 (27.2)	27 (25.5)	42 (28.4)	
Current smoker	51 (20.1)	18 (17.0)	33 (22.3)	
HbA1C (%)	7.7 ± 1.3	7.6 ± 1.2	7.7 ± 1.4	0.517
Glucose (mg/dL)	145.2 ± 46.2	143.1 ± 46.9	146.7 ± 45.8	0.547
SBP (mmHg)	132.2 ± 18.0	131.8 ± 17.7	132.4 ± 18.3	0.768
DBP (mmHg)	74.4 ± 11.9	75.9 ± 10.7	73.4 ± 12.3	0.088
Total cholesterol (mg/dL)	145.5 ± 35.4	151.2 ± 34.2	141.3 ± 35.8	0.028
Triglyceride (mg/dL)	132.7 ± 72.9	135.2 ± 79.1	130.9 ± 68.3	0.641
HDL-cholesterol (mg/dL)	45.5 ± 10.9	46.8 ± 10.5	44.6 ± 11.2	0.122
LDL-cholesterol (mg/dL)	91.3 ± 28.5	95.0 ± 27.7	88.6 ± 28.9	0.074
UACR [*n* (%)]				0.149
Normal (<30 mcg/mg)	168 (66.1)	76 (71.7)	92 (62.2)	
Microalbuminuria (30~300 mcg/mg)	63 (24.8)	20 (18.9)	43 (29.1)	
Albuminuria (>300 mcg/mg)	21 (8.3)	9 (8.5)	12 (8.1)	
Creatinine (mg/dL)	1.0 ± 0.6	1.0 ± 0.7	1.0 ± 0.6	0.800
eGFR (%)	82.5 ± 20.6	84.3 ± 21.4	81.2 ± 19.9	0.237
Carotid IMT (mm)	0.73 ± 0.02	0.69 ± 0.02	0.75 ± 0.01	0.014
Presence of carotid plaque [*n* (%)]	155 (61.0)	15 (14.1)	140 (94.6)	<0.001

*DM, diabetes mellitus; OHA, oral hypoglycemic agent; SBP, systolic blood pressure; DBP, diastolic blood pressure; HDL, high density lipid; LDL, low density lipid; UACR, urine albumin to creatinine ratio; eGFR, estimated glomerular filtration rate; IMT, intima media thickness*.

Regarding the ophthalmologic data, BCVA, DR stage, and OCTA signal strength were not significantly different in terms of subclinical atherosclerosis (Table 2). Whereas, the area, perimeter, and acircularity of FAZ were not different between the two groups, VD around FAZ was significantly more impaired in the subclinical atherosclerosis group (47.1 ± 3.6 vs. 48.3 ± 3.8, *p* = 0.009). While foveal VD in the SCP and DCP was not different between two groups, parafoveal VD in the SCP (45.7 ± 3.6 vs. 47.2 ± 3.7, P = 0.002) and DCP (49.5 ± 3.6 vs. 50.5 ± 3.5, P = 0.044) was significantly reduced in the subclinical atherosclerosis group. There was no significant difference in scan quality in terms of subclinical atherosclerosis to identify the factors associated with presence of the subclinical atherosclerosis, univariate and multivariate logistic regression analyses were conducted including the baseline variables and OCTA parameters. In the univariate analysis (Table 3), old age [*OR* = 1.06 (95% *CI* 1.03–1.09), *p* < 0.001], male sex [*OR* = 2.42 (95% *CI* 1.43–4.09), *p* = 0.001], longer duration of DM [*OR* = 1.04 (95% *CI* 1.01–1.08), *p* = 0.014], and the presence of hypertension [*OR* = 1.70 (95% *CI* 1.02–2.82), *p* = 0.042] were associated with the presence of subclinical atherosclerosis. When all patients were divided into two groups according to DR severity, marginal association was confirmed in the univariate analysis [*OR* = 2.16 (95% *CI* 0.85–5.79), *p* = 0.075]. Among the OCTA parameters, decrease in foveal VD around FAZ [*OR* = 0.91 (95% *CI* 0.85–0.98), *p* = 0.010] and parafoveal VD in SCP [*OR* = 0.89 (95% *CI* 0.83–0.96), *p* = 0.002] and DCP [*OR* = 0.93 (95% *CI* 0.87–1.00), *p* = 0.045] was associated with subclinical atherosclerosis.

**Table 2:** Baseline ophthalmologic characteristics and optical coherence tomography angiography (OCTA) parameters of patients.

	**Total (*n* = 254)**	**Subclinical atherosclerosis (–) (*n* = 106)**	**Subclinical atherosclerosis (+) (*n* = 148)**	***P-*value**
BCVA (LogMAR)	0.07 ± 0.09	0.06 ± 0.08	0.08 ± 0.09	0.144
DR stage [*n* (%)]				0.159
No DR	33 (13.0)	18 (17.0)	15 (10.1)	
Mild NPDR	111 (43.7)	50 (47.2)	61 (41.2)	
Moderate NPDR	56 (22.1)	20 (18.9)	36 (24.3)	
Severe NPDR	42 (16.5)	13 (12.3)	29 (19.6)	
PDR	12 (4.7)	5 (4.7)	7 (4.7)	
FAZ parameters	
Area (mm^2^)	0.38 ± 0.59	0.35 ± 0.11	0.40 ± 0.77	0.486
Perimeter (mm)	2.38 ± 0.43	2.43 ± 0.45	2.35 ± 0.41	0.161
VD around FAZ (%)	47.6 ± 3.7	48.3 ± 3.8	47.1 ± 3.6	0.009
Acircularity	1.16 ± 0.06	1.17 ± 0.07	1.16 ± 0.04	0.087
SCP parameters	
Fovea VD (%)	14.8 ± 5.1	14.5 ± 4.8	14.6 ± 5.4	0.817
Parafovea VD (%)	46.3 ± 3.7	47.2 ± 3.7	45.7 ± 3.6	0.002
DCP parameters	
Fovea VD (%)	27.5 ± 6.5	27.4 ± 6.2	27.7 ± 6.8	0.722
Parafovea VD (%)	49.9 ± 3.7	50.5 ± 3.5	49.5 ± 3.8	0.044
Scan quality	8.3 ± 2.2	8.2 ± 2.2	8.0 ± 2.0	0.075

*BCVA, best corrected visual acuity; DR, diabetic retinopathy; NPDR, non-proliferative diabetic retinopathy; PDR, proliferative diabetic retinopathy; FAZ, foveal avascular zone; VD, vessel density; SCP, superficial capillary plexus; DCP, deep capillary plexus*.

**Table 3:** Factors associated with the presence of subclinical atherosclerosis in patients with type 2 diabetes in univariate logistic analysis.

	**Odds ratio (95% CI)**	***P-*value**
**Demographics**	
Age	1.06 (1.03–1.09)	<0.001
Sex		
Male	2.42 (1.43–4.09)	0.001
Female	1 (Ref)	
Hypertension	1.70 (1.02–2.82)	0.042
DM duration	1.04 (1.01–1.08)	0.014
DM treatment		
OHA only	1 (Ref)	
Insulin	0.78 (0.30–2.05)	0.613
Hyperlipidemia	1.23 (0.95–1.58)	0.165
**Laboratory data**	
HbA1C	1.07 (0.88–1.30)	0.515
Glucose	1.00 (1.00–1.01)	0.546
SBP	1.02 (0.99–1.03)	0.622
DBP	0.98 (0.95–1.04)	0.703
Total cholesterol	0.99 (0.99–1.00)	0.030
Triglyceride	1.00 (1.00–1.00)	0.640
HDL-cholesterol	0.98 (0.96–1.01)	0.123
LDL-cholesterol	0.99 (0.98–1.00)	0.076
UACR	
Normal	1 (Ref)	
Microalbuminuria	1.78 (0.96–3.27)	0.066
Albuminuria	1.10 (0.44–2.75)	0.836
Creatinine	1.06 (0.70–1.59)	0.800
eGFR	0.99 (0.98–1.01)	0.237
**Ophthalmologic data**	
BCVA (LogMAR)	9.03 (0.46–175.68)	0.146
DR stage
No DR-mild NPDR	1 (Ref)	
Worse than moderate NPDR	2.16 (0.85–5.79)	0.075
**OCT angiography parameters**
FAZ parameters
Area (mm^2^)	1.25 (0.61–2.56)	0.547
Perimeter (mm)	0.66 (0.36–1.19)	0.163
VD around FAZ (%)	0.91 (0.85–0.98)	0.010
Acircularity	0.02 (0.00–1.88)	0.092
SCP parameters	
Fovea VD (%)	1.01 (0.96–1.06)	0.816
Parafovea VD (%)	0.89 (0.83–0.96)	0.002
DCP parameters	
Fovea VD (%)	1.01 (0.97–1.05)	0.721
Parafovea VD (%)	0.93 (0.87–1.00)	0.045
Scan quality	0.92 (0.85–1.05)	0.116

*DM, diabetes mellitus; OHA, oral hypoglycemic agent; SBP, systolic blood pressure; DBP, diastolic blood pressure; HDL, high density lipid; LDL, low density lipid; UACR, urine albumin to creatinine ratio; eGFR, estimated glomerular filtration rate; BCVA, best corrected visual acuity; DR, diabetic retinopathy; NPDR, non-proliferative diabetic retinopathy; PDR, proliferative diabetic retinopathy; OCT, optical coherence tomography; FAZ, foveal avascular zone; VD, vessel density; SCP, superficial capillary plexus; DCP, deep capillary plexus*.

**Figure 1:**
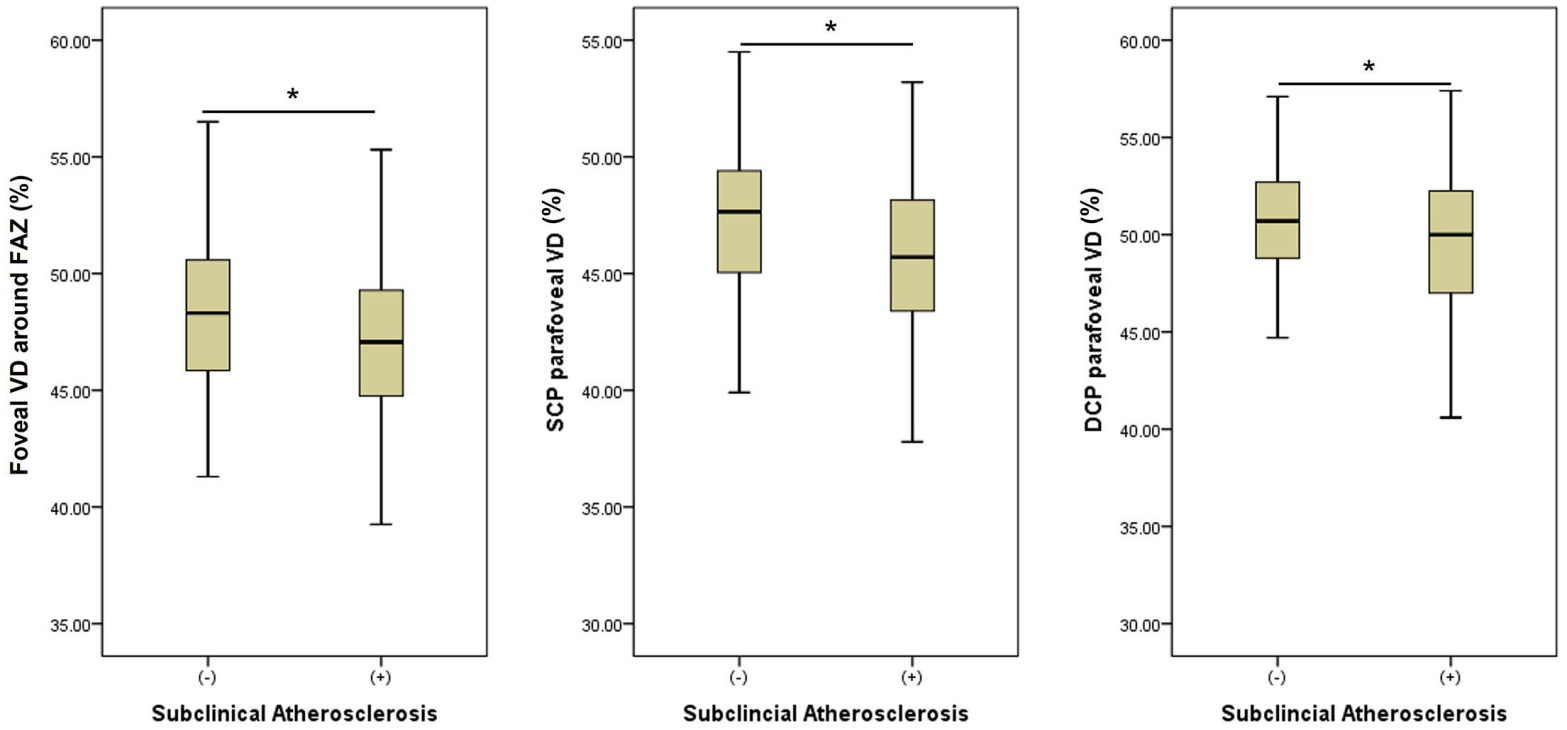
Bar graphs showing the different averages and 95% distributions of the significantly associated optical coherence tomography angiography (OCTA) parameters (foveal vessel density (VD) around foveal avascular zone (FAZ) and parafoveal VD in superficial and deep capillary plexuses (DCP) according to the presence of subclinical atherosclerosis. An asterisk means a statistical significance (*p* < 0.05) between two groups in an independent *t*-test. VD, vessel density; FAZ, foveal avascular zone; SCP, superficial capillary plexus; DCP, deep capillary plexus.

**Figure 2:**
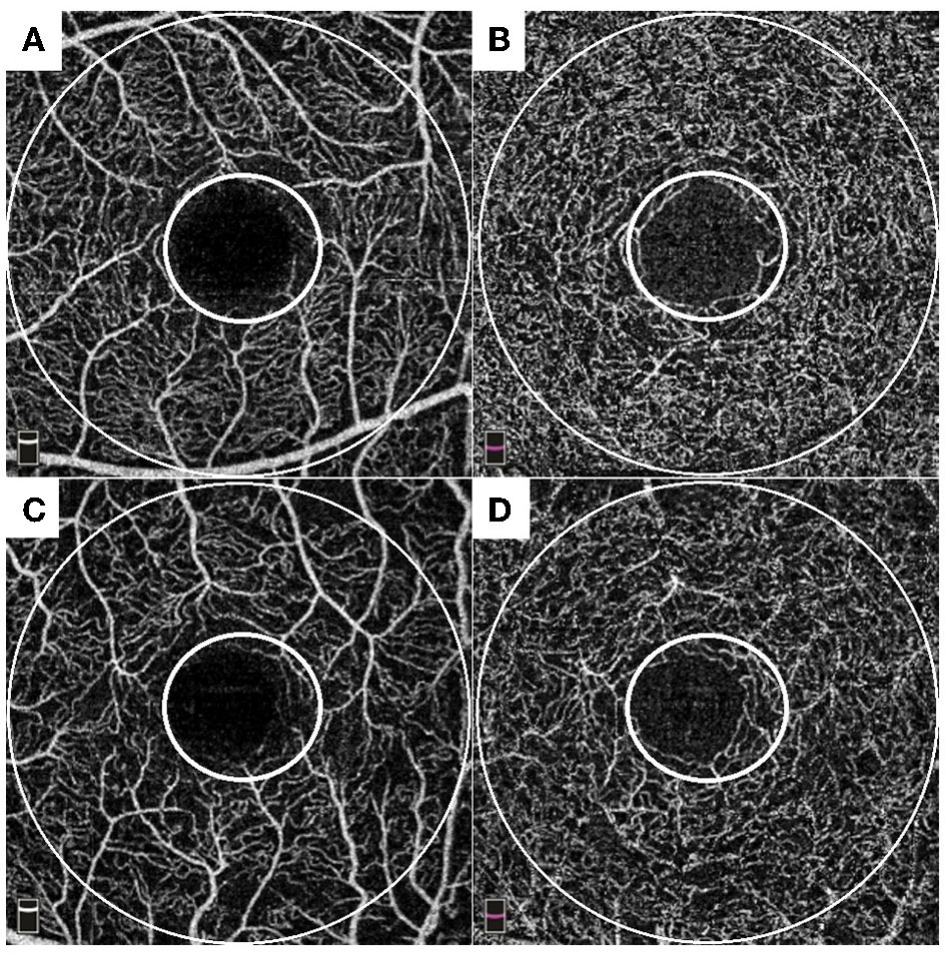
Representative cases of an age-sex matched control **(A,B)** and a patient with subclinical atherosclerosis **(C,D)**. Each of small and large circles denote foveal and parafoveal area. Control: A 56-year- old male patient with 18-years history of diabetes showed 0.63 mm of carotid intima media thickness (IMT) with plaque-free in carotid ultrasonography (US). He had well-preserved foveal VD around foveal avascular zone FAZ (49.7%), parafoveal VD in superficial capillary plexus (49.3%), and parafoveal VD in deep capillary plexus (50.3%) in OCTA. A patient with subclinical atherosclerosis: a 57-year- old male patient with 5-years history of diabetes showed 1.11 mm of carotid IMT with plaque in carotid US. He had impaired foveal VD around FAZ (45.9%), parafoveal VD in superficial capillary plexus (40.8%), and parafoveal VD in deep capillary plexus (46.5%) in OCTA.

We performed three models of multivariate analyses (Table 4) to obviate the confounding effects of the multicollinearity of the OCTA parameters (correlation coefficients >0.8). Old age and male sex were consistently remained as the significant factors for subclinical atherosclerosis (all *p* < 0.05) in all three models. Low foveal VD around FAZ [*OR* = 0.92 (95% *CI* 0.86–1.00), *p* = 0.043], parafoveal VD in both SCP [*OR* = 0.91 (95% *CI* 0.85–0.98), *p* = 0.011], and DCP [*OR* = 0.93 (95% *CI* 0.86–1.00), *p* = 0.046] were significant factors for subclinical atherosclerosis in each of three models. Figure 1 shows the different averages and distributions in the significant OCTA parameters according to the presence of subclinical atherosclerosis. And Figure 2 demonstrated the difference in the foveal and parafoveal capillary vessel density of an age-sex matched control and a patient with subclinical atherosclerosis.

**Table 4:** Factors significantly associated with the presence of subclinical atherosclerosis in patients with type 2 diabetes in multivariate logistic analysis.

	**Model 1 including foveal VD around FAZ**		**Model 2 including SCP Parafovea VD**		**Model 3 including DCP Parafovea VD**	
	**Odds ratio (95% CI)**	***P-*value**	**Odds ratio (95% CI)**	***P-*value**	**Odds ratio (95% CI)**	***P-*value**
Age (year)	1.06 (1.03–1.10)	<0.001	1.06 (1.03–1.09)	0.001	1.06 (1.03–1.10)	<0.001
Male sex	2.67 (1.51–4.75)	0.001	2.77 (1.56–4.93)	0.001	2.77 (1.56–4.90)	<0.001
DM duration (yr)	1.02 (0.98–1.06)	0.456	1.02 (0.98–1.06)	0.425	1.02 (0.98–1.06)	0.409
Hypertension	1.24 (0.71–2.18)	0.448	1.27 (0.72–2.23)	0.412	1.34 (0.76–2.35)	0.311
Foveal VD around FAZ	0.92 (0.86–1.00)	0.043				
SCP Parafovea VD			0.91 (0.84–0.98)	0.011		
DCP Parafovea VD					0.93 (0.86–1.00)	0.046

*Model 1, 2, and 3 contains each of OCTA parameters which showed associations with subclinical atherosclerosis in univariate analyses*.

*DM, diabetes mellitus; VD, vessel density; FAZ, foveal avascular zone; SCP, superficial capillary plexus; DCP, deep capillary plexus*.

## Discussion

This study demonstrates that the decreases in VD of macular microvasculatures were associated and the presence of subclinical atherosclerosis in type 2 DM, suggesting associations between macro- and microvascular diabetes complications. Based on our findings, the alterations of macular microvasculatures in OCTA which are implicative of higher risk of subclinical atherosclerosis, could be used as one of the non-invasive imaging biomarkers for the higher risk of macrovascular diseases which requires careful monitoring.

Our results showing the associations between the carotid disease and retinal vasculatures in diabetes were in line with the previous studies that proved the increased cardiovascular risks in patients with DR. DR is an independent risk factor for carotid plaques, and the severity of carotid atherosclerosis correlates with the severity of microangiopathy. In this study, we could provide stronger evidence for those findings through access to a more sensitive retinal imaging modality than the conventionally used color fundus photography. Morphologic changes assessed by OCTA in DR, i.e., retinal microvasculature abnormalities, such as capillary dropout, reduced capillary VD, tortuous capillary branches, dilated average vascular caliber, FAZ enlargement, and irregular FAZ contour, were present before the beginning of the clinically diagnosed DR and become more obvious as DR progress. As a result, we revealed general reduction in VD in terms of subclinical atherosclerosis.

Interestingly, however, we could not find significant differences in area and contour of FAZ and foveal VD in terms of subclinical atherosclerosis. These differences imply that the overall hemodynamic changes of retinal vasculatures may reflect systemic risk factors related to subclinical atherosclerosis more sensitively, compared with the localized deformation of retinal vessels in FAZ. Moreover, foveal VD which means VD within a fovea centered circle of 1 mm diameter is mostly influenced by the FAZ area. In other words, when the FAZ area is large, the foveal VD is small, and when the FAZ area is small, the foveal VD is large. Therefore, parafoveal VD or VD around FAZ reflects vascular impairment more accurately than foveal VD, which is related to the FAZ area with large individual variability.

Our results showing the close associations between retinal microvasculature obtained by OCTA and diabetic macrovascular complications were in line with those by Drinkwater et al. (12). On the other hand, it is differentiated by the fact that not only carotid stenosis represented by carotid IMT thickening but also carotid plaque, which is a predictor of atherosclerotic cardiovascular diseases. Most of our patients classified as the subclinical atherosclerosis group had carotid plaques without IMT thickening. Moreover, when we evaluated the VD changes of each layer, we noted that parafoveal VDs in both SCP and DCP were all correlated with subclinical atherosclerosis. These results were different from their study which concluded that the decrease VD in only DCP correlates to the increased IMT and the grade of stenosis and VD in SCP did not show significant association with the carotid parameters (17). This difference primarily may be due to the different patient characteristics, particularly in the distribution of DR stages between two studies. While our study included the patients with variable stages of DR (13.1% patients with no DR), the study by Drinkwater et al. (12) mainly included the patients with no DR (83.8% patients with no DR). Since it is widely reported that the vascular changes in DCP occur in the early stage of DR (even before the development of DR) and those in SCP occur in the later stage, patients with no DR or early stage of DR might not have the significant changes in SCP (18). Rather, our data showed that the degree of association between subclinical atherosclerosis and reduction in VD was slightly higher in SCP compared with that of DCP. While metabolic diseases, i.e., diabetes mainly affect DCP with slower blood flow, where toxic materials take longer to contact the blood vessels, arterial diseases, i.e., hypertension, act more on precapillary arterioles where shear stress and oxidative stress work well (19).

The pathogenic mechanism of how carotid diseases associates with retinal microvascular disease is not well-established, although there are several hypotheses. Similar risk factors may contribute to both diseases. In addition, microcirculation damage caused by diabetes serves as the “common soil” for macro- and microangiopathy of diabetes, since diabetic macroangiopathy evolves from the microvascular damage within the major arterial wall (the vasa vasorum) (20). Recent evidence has shown that the vasa vasorum of the major vessels in patients with diabetes undergoes the similar process as the microvascular changes of the DR. Endothelial dysfunction and increase of vascular permeability occur at first, followed by hypoxia, which leads to the angiogenesis and neovascularization. Therefore, this shared vascular pathophysiology proves that microangiopathy and macroangiopathy of the diabetes are not entirely separated entities.

In our analysis, we could not confirm the differences in BP, blood lipid, glucose control level, and treatment thereof, known factors which affect retinal vasculatures, according to the presence of subclinical atherosclerosis. This can be explained by several reasons. First, this study was conducted on patients who had undergone medical treatment for BP, blood lipid, and glucose. The second reason is that the systemic clinical data included in this study were measured on the day of the visit to the internal medicine clinic, not on the exact date of OCTA acquisition. Considering the variability of medical indicators, the possibility that the time difference affected in the lack of associations cannot be excluded.

The present study has some limitations, including its retrospective nature of study design. The other limitation is that our measurements based on a small field of view (3 mm × 3 mm) of OCT angiography, which may not represent the whole retinal circulation. Despite these limitations, this approach could provide the important clinical implications of predicting the systemic status with widely available ocular images captured in a short time. The other strength of this study is that we focused only on patients with no DR or treatment naïve patients with DR to obviate the possible effects of ocular treatments on retinal vasculatures. Since previous studies reported changes in macular vasculatures after laser photocoagulation or intravitreal injections (21, 22), we believe that this point has an importance for the accurate analysis. In addition, to minimize the possible errors in image analysis, we included only patients with good OCTA image quality of scan quality ≥7, proper segmentation without errors, and the removal of projection artifact, which are all major factors that must be carefully considered in an OCTA imaging study. Last, the number of patients was sufficient for the analysis of risk factors for subclinical atherosclerosis.

In conclusion, we found that decreased VD around FAZ and parafoveal VD in OCTA were significantly associated with subclinical atherosclerosis with other risk factors, such as male sex and old age. Non-invasive *in vivo* retinal vascular imaging captured by OCTA could be used to assess DR but also as the early indicator of macrovascular complications, which suggests that diabetic microangiopathy and macroangiopathy may share the common pathophysiology. Therefore, ophthalmologists should keep in mind such close relationship between ocular changes and systemic diseases and consider evaluations for other comorbidities, such as carotid US, when they examine the patients with impaired macular vasculatures.

## Data Availability Statement

The raw data supporting the conclusions of this article will be made available by the authors, without undue reservation.

## Ethics Statement

The studies involving human participants were reviewed and approved by Asan Medical Center IRB. Written informed consent for participation was not required for this study in accordance with the national legislation and the institutional requirements.

## Author Contributions

All authors listed have made a substantial, direct, and intellectual contribution to the work and approved it for publication.

## Funding

This study was supported by a grant from the Technology Innovation Program (or Industrial Strategic Technology Development Program) (1415175064, Development of portable fundus imaging and diagnosis device equipped with artificial intelligence and edge computing) funded by the Ministry of Trade, Industry & Energy (MOTIE, South Korea), and the Asan Institute for Life Sciences (2020IP0103-1), Asan Medical Center, Seoul, South Korea.

## Conflict of Interest

The authors declare that the research was conducted in the absence of any commercial or financial relationships that could be construed as a potential conflict of interest.

## Publisher's Note

All claims expressed in this article are solely those of the authors and do not necessarily represent those of their affiliated organizations, or those of the publisher, the editors and the reviewers. Any product that may be evaluated in this article, or claim that may be made by its manufacturer, is not guaranteed or endorsed by the publisher.
